# Consumers’ Views on the Importance of Specific Front-of-Pack Nutrition Information: A Latent Profile Analysis

**DOI:** 10.3390/nu11051158

**Published:** 2019-05-23

**Authors:** Liyuwork Mitiku Dana, Kathy Chapman, Zenobia Talati, Bridget Kelly, Helen Dixon, Caroline Miller, Simone Pettigrew

**Affiliations:** 1 School of Psychology, Curtin University, Kent St, Bentley, WA 6102, Australia; liyuwork.dana@curtin.edu.au (L.M.D.); zenobia.talati@curtin.edu.au (Z.T.); Helen.Dixon@cancervic.org.au (H.D.); 2 School of Life and Environment Sciences, Faculty of Science, University of Sydney, Camperdown, NSW 2006, Australia; kathyc.kathryn@gmail.com; 3 School of Medicine & Public Health, University of Newcastle, University Drive, Callaghan, NSW 2308, Australia; 4 Early Start, School of Health and Society, University of Wollongong, Northfields Ave, Wollongong, NSW 2522, Australia; bkelly@uow.edu.au; 5 Centre for Behavioural Research in Cancer, Cancer Council Victoria, 615 St Kilda Rd, Melbourne, VIC 3004, Australia; 6 Melbourne School of Psychological Sciences, The University of Melbourne, Melbourne VIC 3010, Australia; 7 Population Health Research Group, South Australian Health and Medical Research Institute, North Terrace, Adelaide, SA 5000, Australia; Caroline.Miller@sahmri.com; 8 School of Public Health, University of Adelaide, Adelaide, SA 5000, Australia

**Keywords:** food labels, front-of-pack labelling, nutrition information panels, nutrients, energy

## Abstract

Nutrition labelling can influence consumers’ assessments of food healthiness and their food choices. However, there is a lack of consensus about the optimal type and amount of nutrition information to provide on food packages. This study analysed consumers’ preferences for front-of-pack information relating to energy and various nutrients (sugar, saturated fat, sodium, fibre, carbohydrate, and protein). The aim was to identify discrete preference segments within the Australian market where the current Health Star Rating front-of-pack labelling system can be displayed with different levels of nutrition information. Adults (*n* = 1558) completed a survey assessing socio-demographics, self-reported nutrition knowledge, diet healthiness, special dietary requirements, and perceived importance of the provision of energy and nutrient information on the front of food packs. Latent profile analysis identified five consumer segments within the sample that ranged from groups exhibiting high levels of interest in various forms of nutrition information to one with very low interest and one with divergent scores according to whether nutrients were perceived as positive or negative for health. The results indicate that different forms of front-of-pack labelling featuring varying degrees of information about energy and specific nutrients are likely to be of interest and use to different market segments.

## 1. Introduction

Eating patterns in many nations are inconsistent with dietary guidelines [1,2,3], which is reflected in the increasing prevalence of diet-related diseases and obesity [4,5,6,7]. Dietary guidelines generally recommend limiting energy-dense, nutrient-poor foods and beverages and encourage daily consumption of a wide variety of foods from the five core food groups (vegetables, fruit, grain cereals, lean meats and alternatives, and dairy foods and alternatives) [8,9,10].

In many countries, home cooking has declined in recent decades due to a number of factors including time constraints, longer working hours, and greater availability of convenience foods [11,12,13]. As a result, there is now a heavy reliance on pre-packaged, ready-to-eat, and ready-to-heat processed foods, which tend to be energy-dense and nutrient-poor [11,14,15]. These trends are leading to excessive intakes of energy and nutrients associated with chronic disease risk, such as salt, saturated fat, and added sugar [16]. For example, it has been estimated that over 75% of energy intake is derived from processed foods in the US [14], and 95% of dietary salt in the United Kingdom comes from processed foods [17]. Globally, excess salt/sodium intake has been attributed to 1.7 million cardiovascular deaths and 4.1 million total deaths each year [18,19].

One way of improving diet quality while satisfying consumers’ desire for convenience is to facilitate healthier choices of packaged foods [20]. Nutrition labelling on packaging is a demonstrated method of improving consumers’ ability to assess product healthiness and encouraging healthier food choices [21,22,23,24,25]. On-pack food labelling systems include the provision of specific information about individual nutrients (e.g., nutrition information panels on the back or side of packs and percent Reference Intakes), the use of symbols on the front of packs that provide an overall indication of product healthiness (e.g., Keyhole, Nutri-score), and variants that combine an overall interpretive symbol with numerical nutrient information (e.g., Multiple Traffic Lights, Health Star Rating) [25].

Studies have consistently linked the use of nutrition label information with a healthier diet (for a review see [26]). Consumers tend to view nutrition information if it is displayed prominently, and as a result, front-of-pack labelling is more likely to be viewed than nutrition declaration panels that are located on the back or side of packs [27,28,29]. Nutrition declaration panels also require the interpretation of numerical information, and as such can be difficult for consumers to understand and use [26,30,31]. Evidence suggests that interpretive nutrition information tends to be more effective than numerical information in enabling consumers to identify healthier foods [23,24,25,32,33,34], and that interpretive front-of-pack food labels are a cost-effective policy initiative to prevent obesity [35]. Requiring the provision of easy-to-interpret nutrition information on the front of food packages has thus been recognised as an important policy action to improve dietary intakes at a population level [25,36].

Most studies examining consumers’ understanding and use of both interpretive and numerical front-of-pack food labels have assessed outcomes in terms of reactions to the entire label, and only a few have explored consumers’ desire for information relating to specific nutrition elements shown within the label. Studies in the latter category have found that of the various types of nutrients, consumers are most likely to compare products on saturated fat and sugar content to assist them in selecting healthier food choices, followed by sodium information (e.g., [34,37]). Related research on consumers’ overall nutrient information preferences (i.e., in general rather than only on the front-of-packs) has found that many consumers value information about individual nutrients, with vitamins and minerals, saturated fat, sugar, and salt being considered important [30,31,38].

There is also little existing evidence about the information needs of those consumers with special nutrition requirements. Access to nutrition information on the front of packs may become increasingly important given the rapid ageing of many populations and the resulting growth in the prevalence of age-related diseases that are associated with poor diet quality (e.g., heart disease, diabetes, and high blood pressure) [39]. Finally, there is little available evidence on how consumers can be segmented according to their desire for nutrient-specific information located on the front of packs. An exception is Jurado and Gracia’s study of Spanish consumers’ perceptions of on-pack claims relating to saturated fat and fibre [40]. Using latent class modelling, they identified two segments of consumers who valued nutritional claims for specific nutrients and a third segment that comprised those who appeared to avoid nutrition claims.

**Figure 1:**
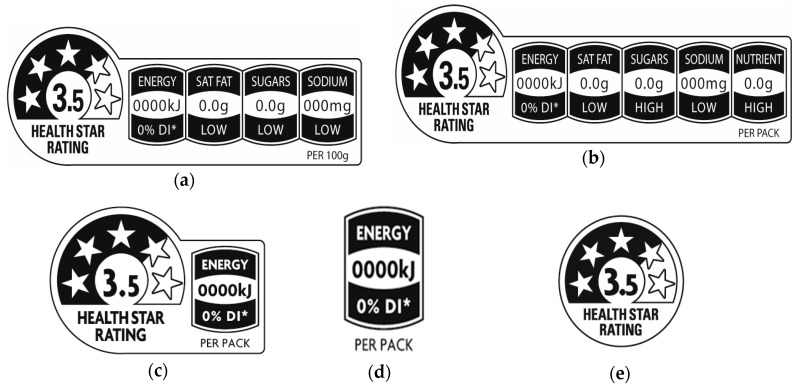
Health Star Rating System (HSR) System labelling options: (**a**) HSR with energy and three nutrient icons; (**b**) HSR with energy and four nutrient icons; (**c**) HSR with energy icon; (**d**) Energy icon only; (**e**) HSR with no additional icon (Source: Food Standards Australia New Zealand [41]).

Segment-level market information can provide useful insights into whether front-of-pack labels exhibiting varying levels of nutrition information may be more appealing and useful to different groups of consumers. This is of substantial relevance in Australia, the context of the present study, because the voluntary Health Star Rating system exists in several different formats that can be selected by food companies for use on their packages [41]. Some of the label variants feature larger or smaller amounts of nutrient-specific information, one provides only energy information, and one has only an interpretive element in the form of the star icon (see Figure 1). In addition to the voluntary Health Star Rating labelling system, the provision of a nutrition declaration panel on the back or side of packs displaying levels of energy, sugar, total fat, saturated fat, sodium, carbohydrate, and protein is mandatory for most foods [42].

Given the lack of research to date relating to preferences for front-of-pack information about specific nutrients and to provide insights into the extent to which various groups of Australians may value the inclusion of this information, the present study aimed to: (i) segment Australian consumers based on the importance of front-of-pack information relating to energy/kilojoules, sugar, saturated fat, sodium/salt, fibre, carbohydrate, and protein; (ii) describe the characteristics of identified groups of consumers; and (iii) consider the implications of the findings for food labelling policy and regulations. The results can assist policy makers and food companies in their deliberations over which forms of the Health Star Rating system to use on products targeting different population segments. In addition, by asking consumers to rate the importance of particular nutrition elements (i.e., energy and specific nutrients) rather than assessing the different forms of the Health Star Rating system, the results are of potential relevance to policy makers in other countries where alternative forms of front-of-pack labels are currently in use or being considered.

## 2. Materials and Methods

This study was part of a larger research project examining consumers’ attitudes to food labelling (protocol detailed in [43]). The study was conducted in accordance with the Declaration of Helsinki, and ethics approval was obtained from the Curtin University Human Research Ethics Committee (Approval number: RDHS-11-15, date of approval: 15 January 2015).

### 2.1. Sample and Study Design

Australians aged 18+ years (*n* = 1558) were recruited via an ISO accredited national web panel provider (PureProfile) to undertake an online survey for a small financial incentive. After providing informed consent, respondents answered questions on their socio-demographic characteristics, the importance of various forms of nutrition information, self-rated nutrition knowledge, perceived healthiness of their diet, and if they were on a special diet. Quotas were set for age and gender to ensure the sample was representative of the Australian population on these variables. Lower socio-economic status (SES) respondents were intentionally over-sampled (representing half of the sample) because they have poorer dietary intakes, higher levels of obesity, and are less likely to read and use nutrition information provided on food labels [44,45], thus making them an especially important group to consider in nutrition information research. Comparisons between the characteristics of the study sample and the Australian adult population are presented in Table 1.

### 2.2. Measures

#### 2.2.1. Socio-Demographics

Respondents were asked to report their age, gender, education, and residential postcodes, along with their weight and height to calculate their Body Mass Index (kg/m^2^). Postcodes were used to identify SES (as per the Australian Bureau of Statistics’ Socio-Economic Indexes for Areas classification [47]) and whether respondents were located in a metropolitan or regional area (as per the Australian Statistical Geography Standard [48]). Respondents were also asked whether they were the main grocery buyer for the household, with response options of ‘*Yes*’, ‘*No*’, and ‘*Share job equally*’.

#### 2.2.2. Perceived Importance of Nutrient Information on Front of Pack

Respondents rated the importance of the availability of front-of-pack nutrition information relating to energy/kilojoules, sugar, saturated fat, salt/sodium, fibre, carbohydrate, and protein content. The questions were worded as “*How important is it to have information on the front of the pack about these nutrients?*” with response options for each nutrient ranging from ‘1-*Very unimportant*’ to ‘5-*Very important*’. The term salt was included in addition to sodium and kilojoules in conjunction with energy to ensure the terminology in the survey questions was familiar to respondents.

#### 2.2.3. Self-Rated Nutrition Knowledge

To assess self-rated nutrition knowledge, respondents were asked “*Compared to most people, I know quite a lot about nutrition*”. Response options ranged from ‘1-*Strongly disagree*’ to ‘5-*Strongly agree*’.

#### 2.2.4. Healthiness of Diet and Special Dietary Requirements

Respondents rated the perceived healthiness of their diet on a 4-point scale (‘1-*I eat a very healthy diet*’ to ‘4-*I eat a very unhealthy diet*’). For analysis purposes, responses to this item were reverse-coded. Respondents also reported whether they had any special dietary requirements associated with high blood pressure, high cholesterol, diabetes, or heart disease (‘*Yes*’, ‘*No*’).

### 2.3. Statistical Analyses

A latent profile analysis approach was used to extract homogeneous segments of respondents [49]. Seven indicator variables were included in the analysis in the form of importance ratings for the seven nutrition elements under investigation (energy/kilojoules, sugar, saturated fat, salt/sodium, fibre, carbohydrate, and protein). The Maximum Likelihood (ML) estimator was used to compute one- to seven-segment models emerging from the data. The best-fitting model was determined based on the statistical indices of Akaike Information Criteria (AIC), Bayesian Information Criteria (BIC), Sample-size Adjusted Bayesian Information Criteria (SABIC), and entropy values. The combination of relatively low AIC, BIC, and SABIC accompanied by a high entropy value is recommended to identify the optimal model solution [50,51,52]. Additional considerations were that the optimal model did not include a segment comprising less than 5% of the sample and was readily interpretable [50,53,54]. Highest posterior probabilities were used to assign individuals to each class, and one-way ANOVAs were then used to identify significant differences in the mean scores of the indicator variables between segments. Bivariate analyses, chi-square tests, or one-way ANOVAs with Bonferroni post hoc tests (depending on the nature of the predictor variables) were then conducted to describe the characteristics of the derived segments. Stata version 15.1 was used for all analyses [55].

## 3. Results

### 3.1. Descriptive Statistics

Across the entire sample, the provision of information on the front of packs about each of the specific nutrients was perceived as at least ‘somewhat important’, with higher scores received for the ‘risk’ nutrients (i.e., sugar, saturated fat, and sodium/salt) relative to the other nutrients. In terms of individual nutrients, respondents rated information on sugar as most important (mean (M) = 3.86, standard deviation (SD) = 0.98), followed by saturated fat (M = 3.84, SD = 0.99), sodium (M = 3.72, SD = 0.99), fibre (M = 3.64, SD = 0.95), and energy (M = 3.62, SD = 0.95). The lowest mean scores were observed for carbohydrate (M = 3.57, SD = 0.94) and protein (M = 3.54, SD = 0.93). The differences in the mean scores were significant except for sugar versus saturated fat, fibre versus energy, and carbohydrate versus protein comparisons (all significant *p*-values were <0.01).

### 3.2. Fit Statistics 

Table 2 shows the statistical indices of the one- to seven-segment models. The values of the AIC, BIC, and SABIC continuously decreased with an increase in the number of latent classes. The entropy values of the four- to seven-segment models were quite similar, while relatively smaller entropy values were found for the one- to three-segment models. The five-segment model was selected as the best-fitting solution based on: (a) the BIC and SABIC improvements from the four- to five-segment models were more substantial than the changes from the successive model comparisons, and substantially higher AIC, BIC, and SABIC values were revealed for the one- to three-segment models; (b) the added segment in the five-segment model provided a different pattern of means and profile shape compared to the classes identified in the four-segment model; (c) the five-segment model did not contain segments that comprised less than 5% of the sample; (d) compared to the five-segment model, the six-segment model did not provide further information on differences in the patterns of the means of the indicator variables (i.e., it did not provide a different latent profile) and the seven-segment model included a segment comprising less than 5% of the sample; and (e) ease of interpretation and parsimony were greater for the five-segment model compared to models with a large number of segments. The entropy value of 0.95 for the five-segment model indicates that 95% of the respondents were classified in the correct latent class, which is higher than the ‘high’ threshold of >0.80 [56]. The average posterior probabilities of being allocated to each profile segment ranged from 0.88 to 0.99, which is higher than the rule of thumb of ≥0.70 that indicates the segments are well separated and precisely classified [54].

### 3.3. Five-Segment Latent Profile Solution

**Figure 2:**
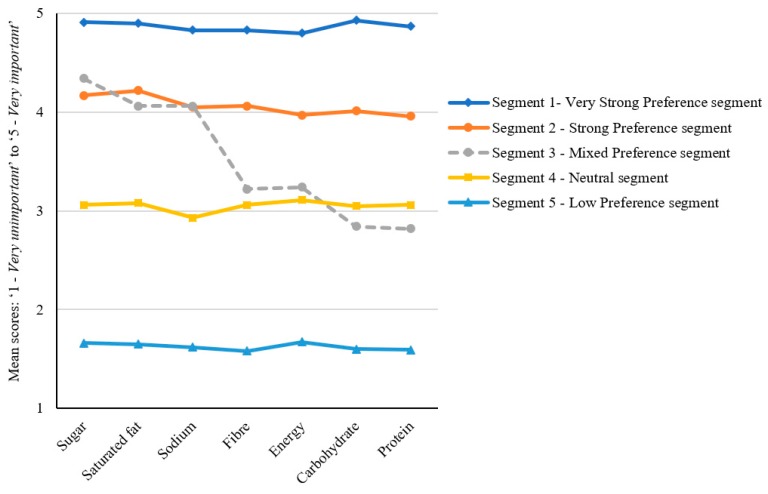
Schematic presentation of the five latent profile segments.

The means of the indicator variables are presented in Table 3, with the distribution pattern illustrated in Figure 2. The derived model yielded a distribution of segments including two groups with a strong stated preference for the provision of information for all the assessed nutrients on the front of food packages (together accounting for 52% of the sample), one group with very low levels of preference across all the nutrients (6%), one group that provided neutral scores across all nutrients (27%), and one demonstrating differing levels of preference depending on the specific type of nutrition information (15%).

Given their relative ratings for the importance of nutrition information being available on the front of packs, the five groups were titled ‘Very Strong Preference’, ‘Strong Preference’, ‘Mixed Preference’, ‘Neutral’, and ‘Low Preference’. There were some distinguishing factors that characterised the groups, although notably there were no significant differences according to gender or BMI.

Overall, the two groups with the strongest preferences for nutrient-specific information had higher proportions of individuals identifying as the main grocery shopper. Relative to the other segments, those in the Very Strong Preference and Strong Preference groups were also more likely to have greater self-rated nutrition knowledge, to have higher perceived diet healthiness, and to report illness-related dietary requirements.

The Mixed Preference segment was unique in two respects. First, this was the only group exhibiting a preference pattern that featured substantial variation between different types of nutrients. Respondents allocated to this group demonstrated a strong desire for information on the front of the pack relating to the ‘risk’ nutrients of sugar, saturated fat, and sodium/salt (average score of 4.15 across these nutrients), but a neutral preference for the other nutrients (average score of 3.03). Second, this was the only segment for which no distinguishing characteristics were evident across the assessed predictor variables relative to the results found for the other segments.

Among respondents allocated to the Neutral segment, average scores for all nutrients were at the mid-point of the 5-point scale. Members of this group tended to be somewhat younger, of higher SES, more likely to have lower perceived diet healthiness, and less likely to be the grocery shopper in the household. Similarly, those in the Low Preference group had relatively low perceived diet healthiness and were more likely than those in most other groups to be of higher SES.

## 4. Discussion

This study analysed Australian consumers’ preferences for front-of-pack nutrition information relating to energy, sugar, saturated fat, sodium, fibre, carbohydrate, and protein. Segmentation analyses were conducted to provide insights into the extent to which different groups of consumers may have varying front-of-pack nutrition information needs and to describe the characteristics of these different market segments. Overall, the risk nutrients of sugar, saturated fat, and sodium were found to be of most interest, which is similar to the results of previous Australian research [34], but different to outcomes from Europe where consumers were found to be most interested in positive nutrients [38]. The Australian response follows prospect theory, whereby consumers attach greater importance to potential loss attributes, in this case risk nutrients, rather than potential gains (nutrients recommended by health authorities) [57].

Of note is that although the provision of information about energy content was rated as at least ‘somewhat important’ by many respondents, it was considered less important than most of the specific nutrients assessed. Previous research indicates that this outcome may reflect a lack of understanding among consumers of the term energy and its important role in determining weight status, with many instead associating it with vitality and positive health benefits rather than as a contributing factor for overconsumption and weight gain [58,59]. This suggests that improving “energy literacy” to assist consumers to better identify high-energy foods is a potential policy action.

The latent profile analysis yielded five distinct segments of consumers based on the perceived importance of front-of-pack information relating to levels of energy, sugar, saturated fat, sodium, fibre, carbohydrate, and protein. Two segments comprising 52% of the sample (the ‘Very Strong Preference’ and ‘Strong Preference’ groups) rated having information on all individual nutrients and energy as important. Consistent with previous research, respondents allocated to these two segments were somewhat more likely to report having special dietary requirements due to a health condition [40]. The segments encompassing those who exhibited neutral and low levels of interest in front-of-pack nutrition information comprised one-third of the total sample (i.e., the ‘Neutral’ and ‘Low Preference’ groups), and were characterised by higher SES. This finding relating to SES is contrary to the outcomes of previous studies showing that those who live in more socially advantaged areas tend to prefer and use more nutrition information [26]. This counter-intuitive finding indicates the need for further research to determine the nature of the relationship between SES and nutrition information preferences, and in particular whether different results are obtained depending on the specified location of the information (i.e., front or back of pack) and the quantity and detail of information provided. Members of the ‘Mixed Preference’ segment, who preferred information on the ‘risk’ nutrients of sugar, saturated fat, and sodium over other nutrients, comprised 15% of the sample. Of note is that unlike for the other segments, there were no clear distinguishing characteristics among this group of consumers despite the comprehensive range of predictor variables included in the study. Further research is therefore needed to determine the factors that may explain why some individuals have a particularly strong interest in negative nutrients and the extent to which this interest manifests in the lower consumption of products that are high in these nutrients.

Various factors are likely to have contributed to the overall preference for detailed nutrient information on the front of food packs found in this study. In the first instance, a voluntary daily intake guide labelling system has existed in Australia for around a decade prior to (and concurrently with) the more recently introduced Health Star Rating system. This means that Australians have been exposed to nutrient-specific information on the front of packs for around 15 years, and as such are likely to have come to expect the availability of such information. Second, research has shown that food products in Australian supermarkets commonly display nutrition content claims [60], which is likely to further consolidate such expectations. Third, consumers can tend to respond in favour of more nutrition information rather than less without considering if they would or could actually use it [61].

The weight of evidence suggests that interpretive front-of-pack food labelling is more effective than nutrient-specific labelling in assisting consumers to identify healthier food products and influencing them to make healthier food choices [22,23,24,25,32,34]. The results of the present study therefore constitute a quandary for policy makers given the identified preference among many respondents for specific nutrition information on the front of packs. However, the present study did not require respondents to choose between nutrient-specific information and interpretive symbols, and instead asked about the absolute (rather than relative) importance of the availability of various types of nutrition information. This information about specific nutrients may be presented on pack in a directive manner, such as through the use of colour-coding or symbols. Further experimental research would be useful to assess how consumers prefer nutrient-specific information to be delivered on the front of packs (e.g., via nutrition claims or nutrition label systems, colour-coded or not) and how such detail may or may not be compatible with the use of highly interpretive labels that provide summary indicators of overall product healthiness, while avoiding the information overload that results in reduced readability and interpretability [62,63]. Such research would be especially useful in the Australian context where the Health Star Rating system currently exists in multiple formats that feature varying levels of nutrient-level information provision. Comparing the relative acceptability and effectiveness of the different formats would assist in guiding future developments in food labelling policy and practice. Preference tracking over time would also be useful for assessing whether consumers’ needs for nutrient-specific information change with population ageing and the growing prevalence of diet-related diseases.

### Study Strengths and Limitations

The primary strength of this study was the use of a novel method of classifying consumers based on their perceived importance of a range of specific types of nutrition information. Latent variable modelling uses goodness-of-fit statistics to identify segments via a probabilistic model approach that is a more advanced person-centred approach compared to traditional cluster analysis. In addition, a broader range of nutrition elements (including both risk and positive nutrients) was included compared to most other work examining consumers’ preferences for nutrient-specific information. The primary limitation was the cross-sectional design, which means only segment membership at a single point in time can be estimated. A longitudinal study using latent transition analysis could address this issue in future research. A further limitation was that respondents were asked about the importance of nutrient information on the front of food packs in general, yet it is conceivable that preferences would vary substantially according to food type (e.g., a ready-to-eat meal versus a yoghurt or bar of chocolate). Finally, the present study did not examine the preferred presentation formats of nutrition-specific information, and this is an important area for future research.

## 5. Conclusions

The majority of the study participants valued the provision of information about a range of nutrients on the front of the food packages, and this was especially evident among those who reported being responsible for household grocery shopping. This suggests that consumers, especially those responsible for household food purchases, would welcome having information on specific individual nutrients routinely provided in the form of front-of-pack nutrition labelling. Interest was strongest for the risk nutrients of sugar, saturated fat, and sodium, indicating that Australian consumers may base their attitudes to foods more on nutrient aversion than nutrient attraction. The provision of nutrient-specific information can facilitate this process, but may not necessarily result in healthier choices given evidence that highly interpretive nutrition information on the front of packs is likely to be most effective in this regard. A tension may therefore exist between preferred versus effective forms of front-of-pack nutrition information. Further research is required to assist in the resolution of this issue. The provision of energy content information was considered of some importance, but was not recognised by respondents as being as important as information relating to risk nutrients, despite research showing energy density and intake play an important role in chronic disease risk and weight gain. More work appears to be needed to determine the best means of educating consumers about the nature and importance of food energy.

## Figures and Tables

**Table 1:** Socio-demographic characteristics of the study sample compared to the Australian adult population (*n* = 1558).

	Sample %	Australian Adult Population [46,47] %
Age (in years)		
18–35	33	33
36–55	33	34
56+	34	33
Gender		
Male	50	49
Female	50	51
Socio-economic status ^1^		
Low	49	40
Medium	32	40
High	19	20

^1^ Socio-economic status determined via the Australian Bureau of Statistics’ Socio-Economic Indexes for Areas (SEIFA) [47]. Lower socio-economic status (SES) respondents were intentionally over-sampled.

**Table 2:** Fit statistics for the one- through seven-segment models for the latent profile analysis.

	Log-Log	Df ^2^	AIC ^3^	BIC ^4^	SABIC ^5^	Entropy	Segments Representing <5% of Total Sample
Segment 1	−15,040.34	14	30,108.68	30,183.59	30,139.11	-	-
Segment 2	−12,811.51	22	25,667.01	25,784.74	25,714.85	0.90	0
Segment 3	−12,213.48	30	24,486.95	24,647.49	24,552.21	0.87	0
Segment 4	−10,971.10	38	22,018.02	22,221.54	22,100.87	0.94	0
Segment 5 ^1^	−10,640.68	46	21,373.36	21,619.51	21,473.47	0.95	0
Segment 6	−10,505.64	54	21,119.28	21,408.24	21,236.84	0.96	0
Segment 7	−10,320.25	62	20,764.50	21,096.27	20,899.51	0.96	1

^1^ The best fitting model. ^2^ df = degrees of freedom. ^3^ AIC = Akaike Information Criteria. ^4^ BIC = Bayesian Information Criteria. ^5^ SABIC = Sample-size Adjusted Bayesian Information Criteria.

**Table 3:** Mean comparisons of profile indicators and predictor variables in the 5-segment latent profile model.

	Total Sample *n* = 1558	Very Strong Preference Segment *n* = 213 13% of Sample	Strong Preference Segment *n* = 609 39% of Sample	Mixed Preference Segment *n* = 231 15% of Sample	Neutral Segment *n* = 419 27% of Sample	Low Preference Segment *n* = 86 6% of Sample	
**Indicator variables ^1^**	**Mean (SD)**	**Mean (SD)**	**Mean (SD)**	**Mean (SD)**	**Mean (SD)**	**Mean (SD)**	**F (*p*-values)**
Sugar	3.86 (0.98) ^1^	4.91 (0.30) ^1,3,a^	4.17 (0.61) ^1,b^	4.34 (0.50)^1,c^	3.06 (0.53) ^1,d^	1.66 (0.64) ^1,e^	870.03 (<0.001)
Saturated fat	3.84 (1.00) ^1^	4.90 (0.40) ^1,3,a^	4.22 (0.61) ^1,b^	4.06 (0.74) ^2,c^	3.08 (0.56) ^1,d^	1.65 (0.66) ^1,e^	691.97 (<0.001)
Sodium	3.71 (0.99) ^2^	4.83 (0.43) ^2,a^	4.05 (0.65) ^2,b^	4.06 (0.66) ^2,b^	2.93 (0.48) ^2,c^	1.62 (0.58) ^1,d^	750.57 (<0.001)
Fibre	3.64 (0.95) ^3^	4.83 (0.42) ^2,a^	4.06 (0.51) ^2,b^	3.22 (0.76) ^3,c^	3.06 (0.50) ^1,d^	1.58 (0.52) ^1,e^	814.42 (<0.001)
Energy	3.62 (0.95) ^3^	4.80 (0.45) ^2,a^	3.97 (0.57) ^3,4,b^	3.24 (0.81) ^3,c^	3.11 (0.57) ^1,c^	1.67 (0.73) ^1,d^	566.07 (0.001)
Carbohydrate	3.57 (0.94) ^4^	4.93 (0.26) ^3,a^	4.01 0.38) ^2,3,b^	2.84 (0.54) ^4,c^	3.05 (0.44) ^1,d^	1.60 (0.52) ^1,e^	1566.04 (<0.001)
Protein	3.54 (0.93) ^4^	4.87 (0.33) ^1,a^	3.96 (0.40) ^4,b^	2.82 (0.52) ^4,c^	3.06 (0.48) ^1,d^	1.59 (0.49) ^1,e^	1322.98 (<0.001)
**Predictor variables**	**Mean (SD)**	**Mean (SD)**	**Mean (SD)**	**Mean (SD)**	**Mean (SD)**	**Mean (SD)**	**F (*p*-values)**
Age (in years)	46.01 (17.35)	49.25 (17.09) ^a^	47.44 (17.36) ^a^	45.84 (16.54) ^a,b^	42.27 (17.15) ^b^	46.51 (18.20) ^a,b^	7.91 (<0.001)
BMI	26.84 (6.24)	27.26 (5.75)	26.68 (6.27)	26.40 (5.19)	27.12 (7.23)	27.01 (5.00)	0.66 (0.621)
Perceived healthiness of diet ^2^	2.96 (0.49)	3.11 (0.50) ^a^	3.02 (0.47) ^a,c^	2.94 (0.41) ^c^	2.81 (0.51) ^b^	2.87 (0.53) ^b,c^	19.23 (<0.001)
Nutritional knowledge ^1^	3.34 (0.85)	3.60 (0.91) ^a^	3.48 (0.82) ^a^	3.20 (0.82) ^b^	3.11 (0.77) ^b^	3.16 (1.07) ^b^	19.70 (<0.001)
	***n* (%)**	***n* (%)**	***n* (%)**	***n* (%)**	***n* (%)**	***n* (%)**	**X^2^ (*p*-values)**
Gender							6.20 (0.184)
Female	782 (50)	114 (54)	318 (52)	119 (52)	193 (46)	38 (44)	
Males	776 (50)	99 (46)	291 (48)	112 (48)	226 (54)	48 (56)	
SES							37.73 (<0.001)
Low	767 (49)	115 (54) ^a^	320 (53) ^a^	112 (48) ^a,b^	180 (43) ^b^	40 (47) ^a,b^	
Medium	492 (32)	65 (31) ^a,b^	196 (32) ^a,b^	85 (37) ^a^	120 (29) ^b^	26 (30) ^a,b^	
High	298 (19)	33 (15) ^a^	92 (15) ^a^	34 (15) ^a^	119 (28) ^b^	20 (23) ^a,b^	
Had a child (ren) (<18 years)	411 (26)	59 (28)	159 (26)	56 (24)	114 (27)	23 (27)	0.92 (0.922)
Grocery buyer							30.12 (<0.001)
Main grocery shopper	1062 (68)	161 (76) ^a^	441 (73) ^a^	145 (63) ^b^	263 (63) ^b^	52 (61) ^b^	
Share shopping	373 (24)	41 (19) ^a^	129 (21) ^a^	71 (31) ^b^	106 (25) ^b^	26 (30) ^b^	
Doesn’t shop	123 (8)	11 (5) ^a^	39 (6) ^a^	15 (6) ^a^	50 (12) ^b^	8 (9) ^a^	
Special dietary requirement							
High blood pressure	172 (11)	35 (16) ^a^	74 (12) ^a^	26 (11) ^a,b^	36 (8) ^b^	1 (1) ^c^	18.18 (0.001)
High cholesterol	169 (11)	19 (14) ^a^	79 (13) ^a^	20 (9) ^a,b^	38 (9) ^a,b^	3 (3) ^b^	11.86 (0.018)
Diabetes	107 (7)	26 (12) ^a^	42 (7) ^c^	8 (3) ^b^	26 (6) ^b,c^	5 (6) ^b,c^	14.12 (0.007)
Heart disease	43 (3)	9 (4)	19 (3)	6 (3)	8 (2)	1 (1)	3.97 (0.410)

Note: sharing the same letter within a row indicates a non-significant difference between segments (*p*-value > 0.05). The *p-*values for the differences in the mean scores of the indicator variables between segments are provided in the Supplementary Material (see Table S1). Sharing the same numbers within a column indicates a non-significant difference within segments on the indicator variables (*p*-value > 0.05). ^1^ Assessed on a 5-point scale. ^2^ Assessed on a 4-point scale. SD: standard deviation.

## Supplementary Materials

The following are available online at https://www.mdpi.com/2072-6643/11/5/1158/s1, Table S1: The p-values of pairwise comparisons of each of the indicator variables between segments.

## References

[1] Haack S.A., Byker C.J. (2014). Recent population adherence to and knowledge of United States federal nutrition guides, 1992–2013: A systematic review. Nutr. Rev..

[2] Raulio S., Ovaskainen M.L., Männistö S., Tapanainen H., Virtanen S., Peltonen M. (2015). Adherence to dietary guidelines in FINDIET2012. Eur. J. Public Health.

[3] Rodríguez-Rodríguez E., Aparicio A., Aranceta-Bartrina J., Gil Á., González-Gross M., Serra-Majem L., Varela-Moreiras G., Ortega R.M. (2017). Low adherence to dietary guidelines in Spain, especially in the overweight/obese population: The ANIBES study. J. Am. Coll. Nutr..

[4] Benziger C.P., Roth G.A., Moran A.E. (2016). The global burden of disease study and the preventable burden of NCD. Glob. Heart.

[5] Imamura F., Micha R., Khatibzadeh S., Fahimi S., Shi P., Powles J., Mozaffarian D. (2015). Dietary quality among men and women in 187 countries in 1990 and 2010: A systematic assessment. Lancet Glob. Health.

[6] Lim S.S., Vos T., Flaxman A.D., Danaei G., Shibuya K., Adair-Rohani H., AlMazroa M.A., Amann M., Anderson H.R., Andrews K.G. (2012). A comparative risk assessment of burden of disease and injury attributable to 67 risk factors and risk factor clusters in 21 regions, 1990–2010: A systematic analysis for the Global Burden of Disease Study 2010. Lancet.

[7] World Health Organization (2017). Obesity and Overweight, fact Sheet. http://www.who.int/news-room/fact-sheets/detail/obesity-and-overweight/.

[8] National Health and Medical Research Council (2013). Australian Dietary Guidelines.

[9] Public Health England (2016). The Eatwell Guide.

[10] U.S. Department of Health and Human Services, U.S. Department of Agriculture, USDA (2016). 2015–2020 Dietary Guidelines for Americans: Dietary guidelines and MyPlate.

[11] Brunner T.A., van der Horst K., Siegrist M. (2010). Convenience food products. Drivers for consumption. Appetite.

[12] Smith L.P., Ng S.W., Popkin B.M. (2014). Resistant to the recession: Low-income adults’ maintenance of cooking and away-from-home eating behaviors during times of economic turbulence. Am. J. Public Health.

[13] Wolfson J.A., Bleich S.N., Smith K.C., Frattaroli S. (2016). What does cooking mean to you? Perceptions of cooking and factors related to cooking behavior. Appetite.

[14] Poti J.M., Mendez M.A., Ng S.W., Popkin B.M. (2015). Is the degree of food processing and convenience linked with the nutritional quality of foods purchased by US households?. Am. J. Clin. Nutr..

[15] Poti J.M., Mendez M.A., Ng S.W., Popkin B.M. (2016). Highly processed and ready-to-eat packaged food and beverage purchases differ by race/ethnicity among US households. J. Nutr..

[16] Steele E.M., Baraldi L.G., da Costa Louzada M.L., Moubarac J.-C., Mozaffarian D., Monteiro C.A. (2016). Ultra-processed foods and added sugars in the US diet: Evidence from a nationally representative cross-sectional study. BMJ Open.

[17] Anderson C.A.M., Appel L.J., Okuda N., Brown I.J., Chan Q., Zhao L., Ueshima H., Kesteloot H., Miura K., Curb J.D. (2010). Dietary sources of sodium in China, Japan, the United Kingdom, and the United States, women and men aged 40 to 59 Years: The INTERMAP study. J. Am. Diet. Assoc..

[18] Forouzanfar M.H., Alexander L., Anderson H.R., Bachman V.F., Biryukov S., Brauer M., Burnett R., Casey D., Coates M.M., Cohen A. (2015). Global, regional, and national comparative risk assessment of 79 behavioural, environmental and occupational, and metabolic risks or clusters of risks in 188 countries, 1990–2013: A systematic analysis for the Global Burden of Disease Study 2013. Lancet.

[19] World Health Organization (2014). Global Status Report on Noncommunicable Diseases 2014.

[20] Tapsell L.C., Neale E.P., Satija A., Hu F.B. (2016). Foods, nutrients, and dietary patterns: Interconnections and implications for dietary guidelines. Adv. Nutr..

[21] Anderson C.L., O’Connor E.L. (2019). The effect of the health star rating on consumer decision-making. Food Qual. Preference.

[22] Cecchini M., Warin L. (2016). Impact of food labelling systems on food choices and eating behaviours: A systematic review and meta-analysis of randomized studies. Obes. Rev..

[23] Egnell M., Talati Z., Hercberg S., Pettigrew S., Julia C. (2018). Objective understanding of front-of-package nutrition labels: An international comparative experimental study across 12 countries. Nutrients.

[24] Talati Z., Norman R., Pettigrew S., Neal B., Kelly B., Dixon H., Ball K., Miller C., Shilton T. (2017). The impact of interpretive and reductive front-of-pack labels on food choice and willingness to pay. Int. J. Behav. Nutr. Phys. Act..

[25] World Cancer Research Fund International (2019). Building Momentum: Lessons on Implementing a rObust Front-Of-Pack Food Label. http://www.wcrf.org/buildingmomentum.

[26] Campos S., Doxey J., Hammond D. (2011). Nutrition labels on pre-packaged foods: A systematic review. Public Health Nutr..

[27] Graham D.J., Heidrick C., Hodgin K. (2015). Nutrition label viewing during a food-selection task: Front-of-package labels vs nutrition facts labels. J. Acad. Nutr. Diet..

[28] Graham D.J., Jeffery R.W. (2011). Location, location, location: Eye-tracking evidence that consumers preferentially view prominently positioned nutrition information. J. Am. Diet. Assoc..

[29] Wills J.M., Schmidt D.B., Pillo-Blocka F., Cairns G. (2009). Exploring global consumer attitudes toward nutrition information on food labels. Nutr. Rev..

[30] Drewnowski A., Moskowitz H., Reisner M., Krieger B. (2010). Testing consumer perception of nutrient content claims using conjoint analysis. Public Health Nutr..

[31] Grunert K.G., Wills J.M., Fernández-Celemín L. (2010). Nutrition knowledge, and use and understanding of nutrition information on food labels among consumers in the UK. Appetite.

[32] Kelly B., Hughes C., Chapman K., Louie J.C.-Y., Dixon H., Crawford J., King L., Daube M., Slevin T. (2009). Consumer testing of the acceptability and effectiveness of front-of-pack food labelling systems for the Australian grocery market. Health Promot. Int..

[33] Morley B., Scully M., Martin J., Niven P., Dixon H., Wakefield M. (2013). What types of nutrition menu labelling lead consumers to select less energy-dense fast food? An experimental study. Appetite.

[34] Watson W., Kelly B., Hector D., Hughes C., King L., Crawford J., Sergeant J., Chapman K. (2014). Can front-of-pack labelling schemes guide healthier food choices? Australian shoppers’ responses to seven labelling formats. Appetite.

[35] Sacks G., Veerman J.L., Moodie M., Swinburn B. (2011). ‘Traffic-light’nutrition labelling and ‘junk-food’tax: A modelled comparison of cost-effectiveness for obesity prevention. Int. J. Obes..

[36] Hawkes C., Jewell J., Allen K. (2013). A food policy package for healthy diets and the prevention of obesity and diet-related non-communicable diseases: The NOURISHING framework. Obes. Rev..

[37] Van Wezemael L., Caputo V., Nayga R.M., Chryssochoidis G., Verbeke W. (2014). European consumer preferences for beef with nutrition and health claims: A multi-country investigation using discrete choice experiments. Food Policy.

[38] Hoefkens C., Verbeke W., Van Camp J. (2011). European consumers’ perceived importance of qualifying and disqualifying nutrients in food choices. Food Qual. Preference.

[39] World Health Organization (2015). World Report on Ageing and Health.

[40] Jurado F., Gracia A. (2017). Does the valuation of nutritional claims differ among consumers? Insights from Spain. Nutrients.

[41] Food Standards Australia New Zealand (2017). Health Star Rating system style guideline (version 5). http://healthstarrating.gov.au/internet/healthstarrating/publishing.nsf/Content/style-guide.

[42] Food Standards Australia New Zealand Nutrition information user guide: Standard 1.2.8—Nutrition information Requirements 2013. https://www.foodstandards.gov.au/code/userguide/Documents/Userguide_Prescribed%20Nutrition%20Information%20Nov%2013%20Dec%202013.pdf/.

[43] Talati Z., Pettigrew S., Dixon H., Neal B., Hughes C., Shilton T., Miller C. (2017). Protocol for a randomized trial assessing consumer evaluations of pre-packaged foods that systematically vary by nutrition information and product attributes. BMC Nutr..

[44] Australian Institute of Health & Welfare, AIHW (2016). Australia’s Health 2016, Chapter 5.

[45] Christoph M.J., Larson N., Laska M.N., Neumark-Sztainer D. (2018). Nutrition facts panels: Who uses them, what do they use, and how does use relate to dietary intake?. J. Acad. Nutr. Diet..

[46] Australian Bureau of Statistics (2017). 31010DO002_201612—Australian Demographic Statistics, Dec 2016: Age distribution, by sex, preliminary–30 June 2016, data cubes.

[47] Australian Bureau of Statistics (2018). 2033.0.55.001—Census of Population and Housing: Socio-Economic Indexes for Areas (SEIFA), Australia, 2016.

[48] Statistics A.B.o., Australian Bureau of Statistics (2013). 1270.0.55.005—Australian Statistical Geography Standard (ASGS): Volume 5—Remoteness structure, July 2011.

[49] Hagenaars J.A.P., McCutcheon A.L. (2002). Applied Latent Class Analysis.

[50] Nylund K.L., Asparouhov T., Muthén B.O. (2007). Deciding on the number of classes in latent class analysis and growth mixture modeling: A Monte Carlo Simulation study. Struct. Equ. Model..

[51] Akaike H. (1974). A new look at the statistical model identification. IEEE Trans. Autom. Control.

[52] DiStefano C., Kamphaus R.W. (2006). Investigating subtypes of child development: A comparison of cluster analysis and latent class cluster analysis in typology creation. Educ. Psychol. Meas..

[53] Marsh H.W., Lüdtke O., Trautwein U., Morin A.J.S. (2009). Classical latent profile analysis of academic self-concept dimensions: Synergy of person- and variable-centered approaches to theoretical models of self-concept. Struct. Equ. Model..

[54] Nagin D.S. (2005). Group-Based Modeling of Development.

[55] StataCorp (2017). Stata Statistical Software: Release 15.1.

[56] Kamata A., Kara Y., Patarapichayatham C., Lan P. (2018). Evaluation of analysis approaches for latent class analysis with auxiliary linear growth model. Front. Psychol..

[57] Kahneman D., Tversky A. (1979). Prospect theory: An analysis of decisions under risk. Econometrica.

[58] Carter O., Mills B., Phan T. (2011). An independent assessment of the Australian food industry’s Daily Intake Guide’energy alone’label. Health Promot. J. Aust..

[59] Watson W.L., Chapman K., King L., Kelly B., Hughes C., Louie J.C.Y., Crawford J., Gill T.P. (2013). How well do Australian shoppers understand energy terms on food labels?. Public Health Nutr..

[60] Hughes C., Wellard L., Lin J., Suen K.L., Chapman K. (2013). Regulating health claims on food labels using nutrient profiling: What will the proposed standard mean in the Australian supermarket?. Public Health Nutr..

[61] Frohlich X. (2017). The informational turn in food politics: The US FDA’s nutrition label as information infrastructure. Soc. Stud. Sci..

[62] Roberto C.A., Bragg M.A., Schwartz M.B., Seamans M.J., Musicus A., Novak N., Brownell K.D. (2012). Facts up front versus traffic light food labels: A randomized controlled trial. Am. J. Prev. Med..

[63] Roberto C.A., Khandpur N. (2014). Improving the design of nutrition labels to promote healthier food choices and reasonable portion sizes. Int. J. Obes..

