# Supplementary material for: Consumers’ Views on the Importance of Specific Front-of-Pack Nutrition Information: A Latent Profile Analysis
Source: Nutrients. 2019 May 23;11(5):1158. doi: 10.3390/nu11051158 (PMC6566345; doi:10.3390/nu11051158)
Supplement: Supplementary file 1 [file nutrients-11-01158-s001.pdf]

Table S1. The *p*-values of pairwise comparisons of each of the indicator variables between segments.

| | **Very Strong Preference segment vs. Strong Preference segment** | **Very Strong Preference segment vs. Mixed Preference segment** | **Very Strong Preference segment vs. Neutral segment** | **Very Strong Preference segment vs. Low Preference segment** | **Strong Preference segment vs. Mixed Preference segment** | **Strong Preference segment vs. Neutral segment** | **Strong Preference segment vs. Low Preference** | **Mixed Preference segment vs. Neutral segment** | **Mixed Preference segment vs. Low Preference** | **Neutral segment vs. Low Preference** |
|---|---|---|---|---|---|---|---|---|---|---|
| **Indicator variables** | ***p-value*** | ***p-value*** | ***p-value*** | ***p-value*** | ***p-value*** | ***p-value*** | ***p-value*** | ***p-value*** | ***p-value*** | ***p-value*** |
| Sugar | <.001 | <.001 | <.001 | <.001 | .001 | <.001 | <.001 | <.001 | <.001 | <.001 |
| Saturated fat | <.001 | <.001 | <.001 | <.001 | .004 | <.001 | <.001 | <.001 | <.001 | <.001 |
| Sodium | <.001 | <.001 | <.001 | <.001 | 1.000 | <.001 | <.001 | <.001 | <.001 | <.001 |
| Fibre | <.001 | <.001 | <.001 | <.001 | <.001 | <.001 | <.001 | .005 | <.001 | <.001 |
| Energy | <.001 | <.001 | <.001 | <.001 | <.001 | <.001 | <.001 | .090 | <.001 | <.001 |
| Carbohydrate | <.001 | <.001 | <.001 | <.001 | <.001 | <.001 | <.001 | <.001 | <.001 | <.001 |
| Protein | <.001 | <.001 | <.001 | <.001 | <.001 | <.001 | <.001 | <.001 | <.001 | <.001 |
